# Kindlin1 regulates microtubule function to ensure normal mitosis

**DOI:** 10.1093/jmcb/mjw009

**Published:** 2016-08-19

**Authors:** Hitesh Patel, Ifigeneia Stavrou, Roshan L. Shrestha, Viji Draviam, Margaret C. Frame, Valerie G. Brunton

**Affiliations:** 1 Edinburgh Cancer Research Centre, Institute of Genetics & Molecular Medicine, University of Edinburgh, Crewe Road South, Edinburgh EH4 2XR, UK; 2 Department of Genetics, University of Cambridge, Downing Street, Cambridge CB2 3EH, UK; 3 Present address: School of Biological and Chemical Sciences, Queen Mary University of London, London, E11 4NS, UK

**Keywords:** Kindlin1, HDAC6, Plk1, microtubules, mitosis

## Abstract

Loss of Kindlin 1 (Kin1) results in the skin blistering disorder Kindler Syndrome (KS), whose symptoms also include skin atrophy and reduced keratinocyte proliferation. Kin1 binds to integrins to modulate their activation and more recently it has been shown to regulate mitotic spindles and cell survival in a Plk1-dependent manner. Here we report that short-term Kin1 deletion in mouse skin results in impaired mitosis, which is associated with reduced acetylated tubulin (ac-tub) levels and cell proliferation. In cells, impaired mitosis and reduced ac-tub levels are also accompanied by reduced microtubule stability, all of which are rescued by HDAC6 inhibition. The ability of Kin1 to regulate HDAC6-dependent cellular ac-tub levels is dependent on its phosphorylation by Plk1. Taken together, these data define a novel role for Kin1 in microtubule acetylation and stability and offer a mechanistic insight into how certain KS phenotypes, such as skin atrophy and reduced cell proliferation, arise.

## Introduction

Loss of Kindlin 1 (Kin1) in humans leads to the skin disorder known as Kindler Syndrome (KS) (Siegel et al., 2003), which is characterized by skin blistering, atrophy, photo-sensitivity, and a predisposition to aggressive squamous cell carcinomas (Siegel et al., 2003; Emanuel et al., 2006; Lai-Cheong et al., 2009). Targeted deletion of Kin1 in the mouse skin leads to similar skin pathologies as those observed in KS patients and reveals a novel role for Kin1 in the regulation of stem cell homeostasis (Rognoni et al., 2014). Kin1 contains a protein:protein interaction FERM domain, which is split by a PH domain that mediates binding to phospholipids. The FERM domain of Kin1 binds to the cytoplasmic tail of integrins and regulates their activation at the cell-substrate boundary (Harburger et al., 2009) and many of the pathologies observed in KS patients and in the mouse model have been attributed to defective integrin regulation. More recently however, we showed that Kin1 localizes to centrosomes and regulates mitotic spindle assembly in an integrin-independent manner (Patel et al., 2013). It binds to and is a substrate of Plk1 and its phosphorylation by Plk1 is critical for fulfilling its role in spindle assembly. To date, the mechanism by which Kin1 regulates mitotic spindle assembly, as well as any potential *in vivo* relevance to KS, remains unclear.

Regulation of microtubule (MT) function is important for mitosis where it is critical for the assembly of a bipolar spindle, alignment of chromosomes, and separation of sister chromatids. The abnormal spindles observed upon Kin1 depletion, i.e. toppled and monopolar, are consistent with defective MT regulation. Therefore, we set out to test whether the role of Kin1 in spindle formation is mediated via the regulation of MTs and whether this is linked to its role during mitosis and in KS. We show that depletion of Kin1 in cells results in reduced MT stability, which is accompanied by reduced ac-tub levels, impaired bipolar spindle formation, and an increased incidence of toppled spindles and lagging chromatids. The acetylation of MTs is a well-established readout of their stability (Piperno et al., 1987) with the cytoplasmic class II histone deacetylase 6, HDAC6, being a critical regulator of this process (Matsuyama et al., 2002). These cellular defects induced upon Kin1 depletion in cells can be rescued by HDAC6 inhibition. Furthermore, Kin1 regulation of HDAC6 is dependent on phosphorylation by Plk1. Finally, Kin1 deletion in the mouse skin results in an increased incidence of toppled spindles, reduced ac-tub, and reduced cell proliferation. These mitotic defects induced by Kin1 deletion are consistent with reduced MT stability and define for the first time a pathway linking Plk1-dependent phosphorylation of Kin1 to the HDAC6 regulation of MTs and mitotic spindle formation.

## Results

### Kin1 deletion in vivo results in abnormal mitosis, reduced acetylated tubulin, and decreased proliferation

KS patients have loss-of-function Kin1 mutations that lead to a number of skin abnormalities, including skin atrophy and reduced cell proliferation. A number of mechanisms may be responsible, including defective cell division. We have recently shown that failure of Plk1 to phosphorylate Kin1 results in cells undergoing defective mitosis, in the form of an increased incidence of toppled and monopolar spindles, leading to cell death (Patel et al., 2013).

To model this in the mouse, we generated a mouse in which exons 4 and 5 of Kin1 were floxed (Kin1^fl/fl^). This allowed us to specifically delete Kin1 in the epidermis by interbreeding Kin1^fl/fl^ mice with K14CreER^T2^ mice in which Cre recombinase activity is under control of the keratin 14 promoter (Li et al., 2000) and 4-hydroxytamoxifen (4OHT). Treatment of the subsequent progeny (K14Cre Kin1^fl/fl^) with 4OHT resulted in deletion of Kin1 in the epidermis after 10 days (Supplementary Figure S1A).

**Figure 1:**
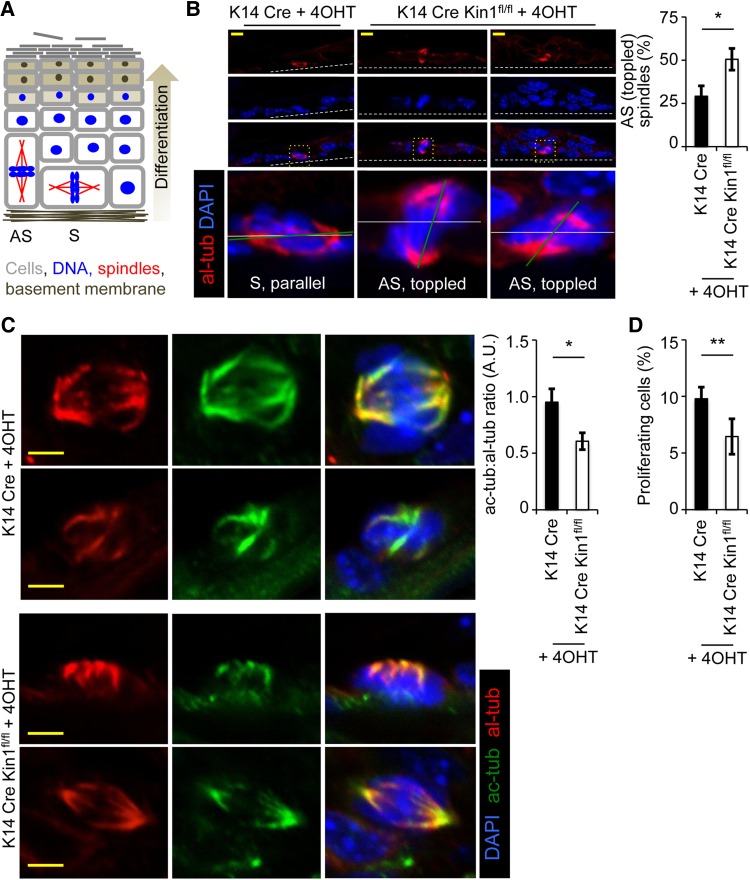
Kin1 deletion *in vivo* results in abnormal mitosis, decreased ac-tub, and decreased cell proliferation. (**A**) Schematic representation of asymmetrical (AS) and symmetrical (S) cell division in the epidermis (adapted from Poulson and Lechler, 2010). Analysis of control (K14Cre) and Kin1-deleted (K14Cre Kin1^fl/fl^) mouse epidermis. (**B**) Representative images (left) of mitotic cells and quantification of asymmetrical spindles (right). (**C**) Representative images (left) of al-tub and ac-tub and quantification (right). (**D**) Percentage of phospho-histone H3-positive cells. In all graphs shown, *n* ≥ 3, error bars are ± SEM. *P*-values, *t*-test, *<0.05 and **<0.01.

In the epidermis, only the basal layer of cells undergoes cell division and these cells do so either symmetrically or asymmetrically (Figure 1A) (Lechler and Fuchs, 2005; Poulson and Lechler, 2010). Symmetrical cell division leads to two basal proliferative cells, while asymmetrical cell division leads to one basal proliferative cell and one non-proliferative cell that goes on to terminally differentiate. Symmetrical or asymmetrical cell division produces bipolar spindles that form parallel to their substrate or spindles that are toppled and form perpendicular/off-parallel to their substrate, respectively. 4OHT-treated dorsal skin sections from K14Cre Kin1^fl/fl^ and control K14Cre mice were stained with alpla-tubulin (al-tub) and acetylated-tubulin (ac-tub) to mark the mitotic spindle, which showed that in Kin1-deleted skin, the incidence of toppled spindles (∼50%) relative to the basement membrane was significantly increased compared with controls (∼25%) (Figure 1B, representative images left and quantification right). Cells were scored as toppled where the plane of the spindles (green line) deviated by ≥45° relative to the plane of the basement membrane (white line). Initial visualization and subsequent quantification of ac-tub levels in mitotic spindles revealed that the ac-tub:al-tub ratio in Kin1-deleted cells was consistently ∼45% lower than that in control cells (Figure 1C, representative images left and quantification right). A potential consequence of increased toppled cell division over time is reduced proliferation due to a lower number of basal proliferative cells. To test this, we scored the number of cells that stained positively for the proliferation marker phospho-Histone H3 (Serine 10) in the basal layer of the epidermis (Figure 1D). The percentage of proliferating cells was significantly lower in the Kin1-deleted epidermis than in controls.

### Kin1 regulates mitosis in an HDAC6-dependent manner

**Figure 2:**
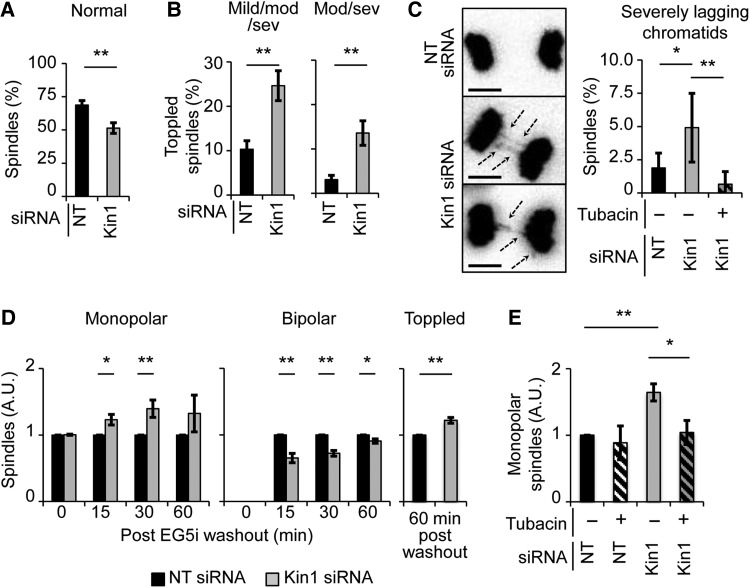
Kin1 regulates mitosis in an HDAC6-dependent manner. (**A–C**) Analysis of NT and Kin1 siRNA-treated mitotic cells using live cell imaging of His2B:DsRed. For control and Kin1 depleted conditions, >250 mitotic cells were observed; for the Kin1 depleted + tubacin condition, >150 mitotic cells were observed. (**A**) Percentage of cells undergoing normal mitosis. (**B**) Percentage of cells undergoing mildly, moderately, or severely toppled mitosis (left) and undergoing moderately or severely toppled mitosis (right). (**C**) Representative images (left) and quantification (right) of lagging chromosomes. (**D**) Quantification of monopolar (left), bipolar (middle), and toppled (right) spindles after release from EG5 inhibition in NT and Kin1 siRNA-treated cells. (**E**) Quantification of monopolar spindles at 30 min post-EG5i washout ± tubacin. For **D** and **E**, >250 mitotic cells were observed. In all graphs shown, *n* ≥ 3, error bars are ± SEM. *P*-values, *t*-test, *<0.05 and **<0.01.

To analyze in more detail the dynamic and temporal nature of the mitotic spindle defects observed *in vivo*, we made use of MDA-MB-231 breast cancer cells that we had previously used to establish the role of Kin1 in mitosis (Patel et al., 2013). Cells stably expressing Histone H2B fused to DsRed (HisH2B:DsRed) to mark DNA were used to follow mitosis using live cell imaging. Though the principle component of mitotic spindles, MTs, are not directly observed, HisH2B:DsRed allows us to infer the position of the spindle as chromosomes condense, align, and separate. Cells normally undergo mitosis with their spindle in the same plane as their substratum. By scoring the plane of sister chromatid separation at anaphase onset, we can infer how well the spindle was aligned to the plane of the substratum. Compared with non-targeting (NT) siRNA-treated (control) cells (∼70%), the percentage of Kin1 siRNA-treated (Kin1 depleted) cells (∼50%) undergoing normal mitosis was significantly reduced (Figure 2A). We found that in Kin1 depleted cells, the incidence of toppled spindles (∼25%) (with respect to the substratum) was increased relative to control cells (∼10%) (Figure 2B, left; Supplementary Movies S1 (control) and S2 (Kin1 depleted)). Furthermore, spindles scored as moderately and severely toppled, where one of the daughter cells failed to adhere for greater than 20 min or at all, were significantly more in Kin1 depleted cells (∼14%) compared with control cells (∼3%) (Figure 2B, right).

Other parameters scored included the length of mitosis, incidence of multipolar spindles, and incidence of lagging chromatids. The length of mitosis (Supplementary Figure S1B) was not significantly different between Kin1 depleted and control cells, and nor was the incidence of multipolar spindles (Supplementary Figure S1C). However, there was a significant increase (2-fold) in the number of mitotic cells that showed severely lagging chromatids at anaphase following Kin1 depletion (Figure 2C, representative images left and quantification right; Supplementary Movies S1 (control) and S3 (Kin1 depleted)). Lagging chromatids can arise from defects in MT function/stability. As we had observed a reduction in ac-tub, a marker of MT stability, upon Kin1 deletion *in vivo* (Figure 1C), we asked whether modulation of ac-tub levels could rescue the lagging chromatid phenotype. The key regulator of ac-tub in cells is HDAC6 (Matsuyama et al., 2002), and tubacin, a widely used and specific inhibitor of HDAC6, prevented the increased incidence of lagging chromatids in Kin1 depleted cells (Figure 2C).

To further define the link between Kin1 and HDAC6 during mitosis, we carried out an assay that more specifically addresses bipolar spindle formation. EG5 is a mitotic kinesin and its inhibition results in the mitotic arrest of cells in a monopolar spindle state (Mayer et al., 1999) without directly affecting MT function/stability. Asynchronous cells were treated with EG5 inhibitor (EG5i) for 2 h and fixed immediately or after EG5i washout for 15, 30, or 60 min. The number of monopolar, bipolar, or toppled mitotic spindles were then scored and plotted for each time point. The percentage of monopolar and bipolar spindles for control cells after EG5i washout is shown in Supplementary Figure S1D. Relative to control cells, Kin1 depletion resulted in an increased number of mitotic cells that persist in the monopolar state and resulted in cells that fail to form a clear bipolar spindle at 15 and 30 min after EG5i washout (Figure 2D). At 60 min post-washout, the number of monopolar spindles observed was not significantly different in control and Kin1 depleted cells. The number of bipolar spindles in the Kin1 depleted cells though was still significantly lower. The incidence of toppled spindles, which showed no difference between Kin1 depleted and control cells at 0, 15, and 30 min post-washout, showed a significant difference at 60 min. These data further support a role for Kin1 in bipolar spindle assembly and spindle orientation—two of the key roles of mitotic MTs.

To determine whether the persisting monopolar spindle phenotype induced by Kin1 depletion is dependent on HDAC6 activity, asynchronous cells were treated with EG5i ± tubacin for 2 h and then released for 30 min ± tubacin (Figure 2E). As observed above, Kin1 depletion led to an increased number of monopolar spindles at 30 min post-EG5i washout. The presence of tubacin rescued the incidence of monopolar spindles observed in Kin1 depleted cells to levels seen in control cells. Thus, the persisting monopolar spindle phenotype induced by Kin1 depletion is also dependent on HDAC6 activity. Expectedly, the ac-tub levels were reduced at 30 min post-EG5i washout in Kin1 depleted cells relative to control (Supplementary Figure S1E).

### Kin1 regulates ac-tub levels in an HDAC6-dependent manner

**Figure 3:**
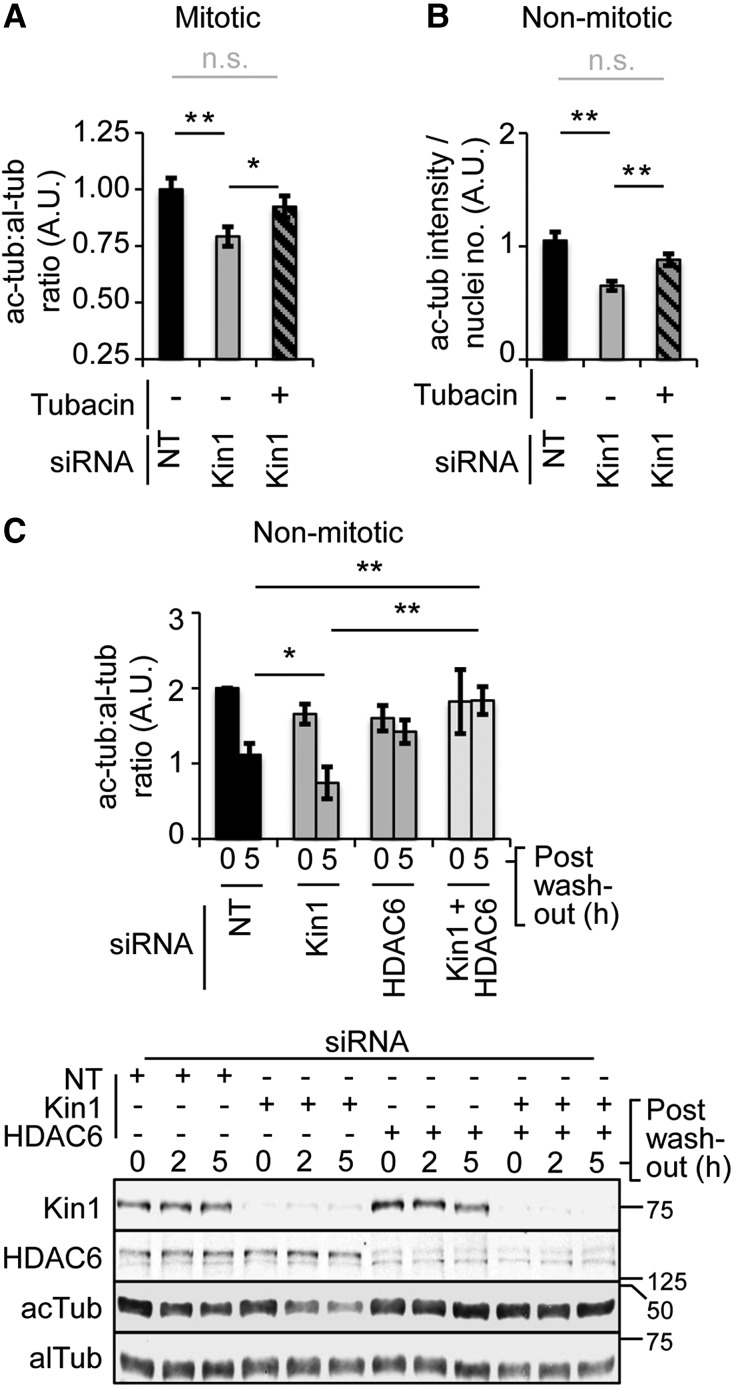
Kin1 regulates ac-tub levels in an HDAC6-dependent manner. (**A**) al-tub and ac-tub levels were determined in NT and Kin1 siRNA ± tubacin-treated mitotic cells by quantification of immunofluorescence images. The double thymidine block and release protocol was used to enrich for mitotic cells. (**B**) ac-tub levels in non-mitotic cells were determined 45 min after disruption of the MT cytoskeleton in MT regrowth assays by quantification of immunofluorescence images in NT or Kin1 siRNA ± tubacin-treated cells. For **A** and **B**, >250 cells were measured. (**C**) ac-tub and al-tub levels determined 0 and 5 h after tubacin washout by quantification of western analysis in cells treated with siRNAs as indicated (top). Representative western blots from which al-tub and ac-tub levels were determined and siRNA depletion confirmed are shown (bottom). In all graphs shown, *n* ≥ 3, error bars are ±SEM. *P*-values, *t*-test, *<0.05, **<0.01, n.s. = not significant.

Data presented above show that depletion of Kin1 *in vivo* (Figure 1C) and in cells (Supplementary Figure S1E) results in reduced ac-tub levels. Moreover, the Kin1 depletion-induced mitotic defects in cells could be rescued by inhibition of HDAC6 (Figure 2C and E). The primary role of HDAC6 is to regulate ac-tub levels in cells, and thus we next tested whether the reduction of ac-tub levels in Kin1 depleted cells is dependent on HDAC6. There was an ∼25% reduction in ac-tub levels in Kin1 depleted mitotic cells compared with control mitotic cells (Figure 3A). Tubacin treatment significantly rescued ac-tub levels in Kin1 depleted mitotic cells to ∼91% of control levels. We next determined whether Kin1 regulation of ac-tub levels also occurred in non-mitotic cells. Kin1 depletion resulted in an ∼40% reduction in ac-tub levels relative to control and the reduction was again rescued by the presence of tubacin to ∼17% of control levels in non-mitotic cells (Figure 3B, quantification; Supplementary Figure S1F, representative images). Tubacin washout assays were then carried out to determine the dynamics of ac-tub turnover. Importantly, in these assays we also used siRNA directed against HDAC6 to complement the previous experiments that had used tubacin to pharmacologically inhibit HDAC6 activity (Figure 3C, quantification above and representative blots below). siRNA (NT, Kin1, HDAC6, and Kin1+HDAC6)-treated cells were incubated with tubacin for 16–20 h to inhibit HDAC6 and allow ac-tub to reach maximal levels. Tubacin was then washed out and ac-tub levels were determined at 0, 2, and 5 h post-washout (Figure 3C, quantification for 0 and 5 h shown for clarity). At 5 h post-tubacin washout, Kin1 depleted cells had significantly lower levels of ac-tub compared with control cells, indicating that Kin1 can regulate the turnover of ac-tub. Concomitant depletion of Kin1 and HDAC6 or HDAC6 alone using siRNA resulted in cells being unable to reduce their ac-tub levels, indicating that HDAC6 is responsible for the reduction of the accumulated ac-tub levels seen in both control and Kin1 depleted cells. Furthermore, in Kin1 depleted cells, there was no significant change in total HDAC6 levels, suggesting that Kin1 regulates the activity of HDAC6.

### Kin1 regulates MT stability

**Figure 4:**
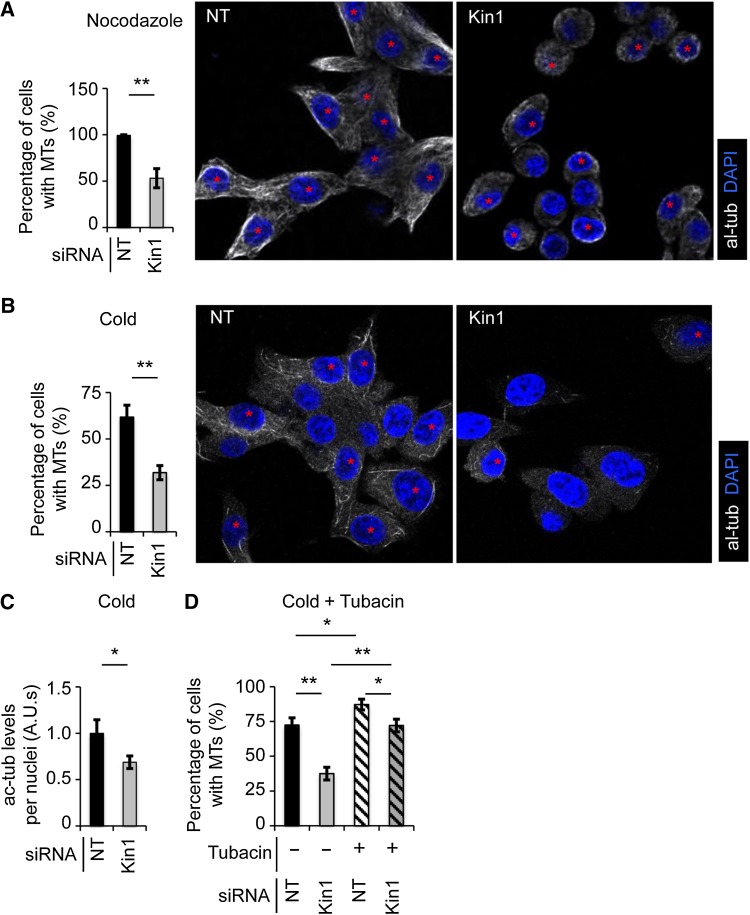
Kin1 regulates MT stability. Representative images (right) show the presence of multiple robust MTs in cells (red asterisk) after nocodazole (**A**) or cold (**B**) treatment, quantified as a percentage of the total number of cells (left). (**C**) ac-tub levels were determined in NT and Kin1 siRNA-treated cells after cold treatment. (**D**) The presence of multiple and robust MTs after cold treatment ± tubacin in cells, quantified as a percentage of the total number of cells. In all graphs shown, *n* ≥ 3, >250 cells were scored and error bars are ±SEM. *P*-values, *t*-test, *<0.05 and **<0.01.

Ac-tub is a well-established marker of more stable MTs in cells (Piperno et al., 1987). As we have shown reduced ac-tub levels *in vivo* and in cells where Kin1 has been depleted, we next asked if it was also associated with reduced MT stability. Two independent approaches were used to test this. Control and Kin1 depleted cells at G1/S were exposed to conditions that impaired the MT cytoskeleton integrity by either 30 min treatment with 0.5 µM nocodazole (De Clerck and De Brabander, 1977) (Figure 4A) or 20 min at 4°C (Behnke and Forer, 1967) (Figure 4B). Both treatments are well-established methods for gauging MT stability and both resulted in Kin1 depleted cells showing ∼50% reduction in the presence of robust stable MTs relative to control cells. As seen with previous assays where we depleted Kin1 in cells, disruption of MTs (by cold treatment) resulted in reduced levels of ac-tub relative to control cells, demonstrating that ac-tubulin levels are linked to MT stability upon Kin1 depletion (Figure 4C). Finally, to determine whether the reduced MT stability seen in the Kin1 depleted cells was dependent on HDAC6 activity, cells were treated with tubacin to inhibit HDAC6 activity in the presence or absence of Kin1 (Figure 4D). Tubacin treatment increased MT stability in NT siRNA-treated control cells and restored MT stability in Kin1 depleted cells to levels observed in control NT siRNA-treated cells (Figure 4D). Thus, Kin1 regulates MT stability in a predominantly HDAC6-dependent manner.

### Kin1 and HDAC6 co-localize and their binding is dependent on Plk1 activity and MT stability

**Figure 5:**
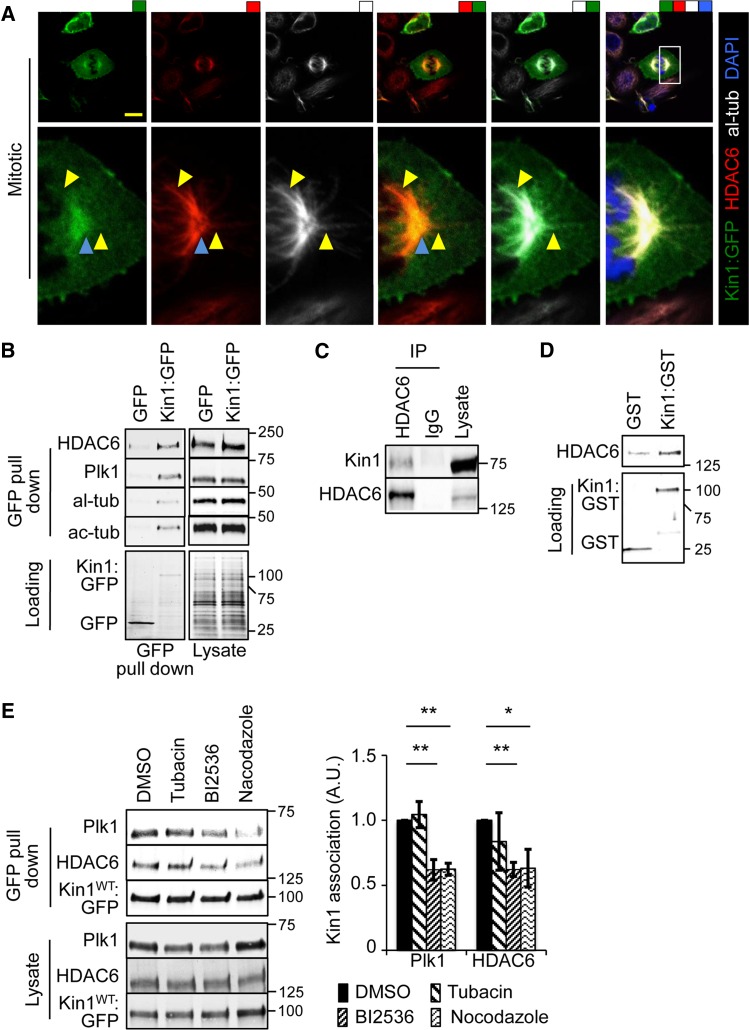
Kin1 localizes with HDAC6 at centrosomes and their association is dependent on Plk1 activity and MTs. (**A**) Kin1:GFP co-localizes with al-tub and HDAC6 at centrosomes (blue arrowheads) and along MT (yellow arrowheads). (**B**) GFP pull downs from lysates of cells expressing Kin1:GFP or GFP were probed with the indicated antibodies. (**C**) Lysates were immunoprecipitated with anti-HDAC6 antibody and probed with Kin1 and HDAC6 antibodies. (**D**) *In vitro* binding assay of recombinant HDAC6 with either GST:Kin1 or GST. (**E**) GFP pull downs from lysate of cells expressing Kin1:GFP in the presence of DMSO, tubacin, BI2536, or nocodazole. In all graphs shown, *n* ≥ 3, error bars are ±SEM. *P*-values, *t*-test, *<0.05 and **<0.01.

We have shown that Kin1 regulates ac-tub levels in cells via HDAC6. This is true in both non-mitotic and mitotic cells. To determine where and how this might be achieved, we carried out co-localization and binding studies. In mitotic cells, Kin1:GFP co-localized with HDAC6 predominantly at centrosomes and occasionally along MTs (Figure 5A). A further example of Kin1:GFP localization at centrosomes and along MTs is shown in Supplementary Figure S2A. Kin1:GFP localization along MTs occurred on filaments extending from the centrosome toward both plasma membrane and chromosomes. Kin1:GFP localization at the centrosomes and along MTs was also observed in non-mitotic cells, suggesting a wider role for this phenomenon than just during mitosis (Supplementary Figure S2B).

GFP pull downs were carried out in Kin1:GFP- and GFP-expressing cells and showed that HDAC6 specifically associated with Kin1:GFP but not GFP (Figure 5B). Our previous study had shown that Kin1 co-localized and interacted with Plk1 at centrosomes (Patel et al., 2013) and co-localization data presented here (Figure 5A) suggest that Kin1 may also associate with tubulin. We confirmed that Plk1, al-tub, and ac-tub were also present in the complex that was pulled down with Kin1:GFP and HDAC6 (Figure 5B).

To test for the specificity of Kin1:GFP binding, we looked for binding to other potential kinases that might phosphorylate Kin1. ELM software (http://elm.eu.org) was used to identify such candidates for the N-terminus of Kin1. Specific consensus sites for phosphorylation by kinases p38 and PKAc (Supplementary Figure S2C) were amongst the candidates and neither was pulled down with Kin:GFP, suggesting that there is a level of specificity in binding to Kin1:GFP (Supplementary Figure S2D).

To confirm the interaction of Kin1 with both HDAC6 and tubulin, we used anti-HDAC6, anti-al-tub, and anti-ac-tub specific antibodies and found that HDAC6 (Figure 5C) and both al-tub and ac-tub (Supplementary Figure S3A) were able to associate with endogenous Kin1. In addition, full-length recombinant HDAC6 was preferentially pulled down with GST fused to Kin1 (GST:Kin1) compared with GST alone, indicating that the association between Kin1 and HDAC6 is direct (Figure 5D). These data indicate that Kin1 associates and co-localizes with HDAC6 and tubulin. Kin1 association with HDAC6 is likely direct, as recombinant proteins show specific binding to each other.

To gain an insight into how the Kin1–HDAC6–Plk1–MT complex is regulated, we used specific inhibitors to target either HDAC6 (tubacin), Plk1 (BI2536), or the MT cytoskeleton (nocodazole). GFP pull downs were used to isolate Kin1:GFP and associated proteins in the presence or absence of the inhibitors (Figure 5E). Inhibition of HDAC6 had no effect on the association between either Plk1 or HDAC6 and Kin1:GFP. However, inhibition of Plk1 activity significantly reduced the association of both Plk1 and HDAC6 with Kin1:GFP. Disruption of the MT cytoskeleton resulted in a similar reduction of association of Plk1 and HDAC6 with Kin1:GFP.

### Plk1 phosphorylation of Kin1 modulates its ability to regulate ac-tub levels via HDAC6

**Figure 6:**
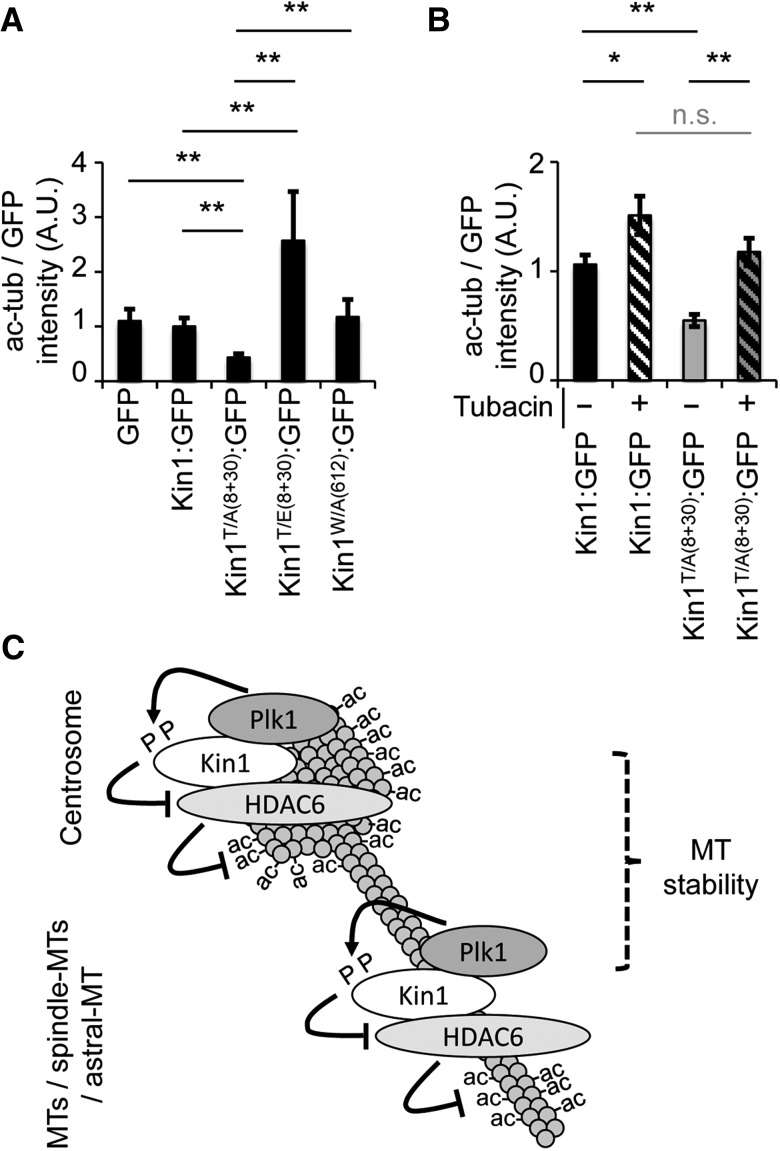
Plk1 phosphorylation of Kin1 modulates its ability to regulate ac-tub levels via HDAC6. (**A** and **B**) Ac-tub levels were determined 45 min after disruption of the MT cytoskeleton in MT regrowth assays in cells expressing GFP or GFP fused to indicated Kin1 mutants (**A**) ±tubacin (**B**). (**C**) A simplified schematic summarizes the interplay between Kin1, Plk1, and HDAC6 in their regulation of MT acetylation and stability. In all graphs shown, *n* ≥ 3, >250 cells were measured and error bars are ±SEM. *P*-values, *t*-test, *<0.05 and **<0.01.

We have previously shown that Kin1 binds to and is phosphorylated by Plk1 on threonines 8 and 30 (T8 and T30), which is required for its ability to regulate mitotic spindles (Patel et al., 2013). Thus, we next asked whether Plk1 phosphorylation of Kin1 (on T8 and T30) is also involved in the ability of Kin1 to regulate ac-tub levels. As Kin1 is primarily known to modulate adhesion through binding and regulating integrin activation, we also asked whether this was involved in the regulation of ac-tub levels. GFP, wild-type (Kin1:GFP), non-phosphorylatable (Kin1^T/A(8+30)^:GFP), phospho-mimicking (Kin1^T/E(8+30)^:GFP), and non-integrin binding (Kin1^W/A(612)^:GFP) forms of Kin1 were expressed in cells and the cellular ac-tub levels were determined (Figure 6A). Relative to ac-tub levels observed in cells expressing GFP or Kin1:GFP, Kin1^T/A(8+30)^:GFP-expressing cells showed an ∼50% decrease in ac-tub levels. In contrast, expression of Kin1^T/E(8+30)^:GFP resulted in an ∼250% increase in ac-tub levels compared with Kin1:GFP-expressing cells. The levels of exogenously expressed Kin1:GFP, Kin1^T/A(8+30)^:GFP, and Kin1^T/E(8+30)^:GFP were similar, as measured by fluorescence intensity in cells (Supplementary Figure S3B). Expression of the integrin binding mutant (Kin1^W/A(612)^:GFP) had no effect on ac-tub levels, indicating that regulation of ac-tub levels by Kin1 is independent of its ability to bind integrins. The reduction in ac-tub levels upon expression of Kin1^T/A(8+30)^:GFP, coupled with our previously published observation that it is also unable to rescue the Kin1 depletion-induced abnormal spindle phenotype, links the ability of Kin1 to regulate ac-tub levels with Kin1 phosphorylation by Plk1 and spindle formation.

We next tested whether the difference in the levels of ac-tub observed upon expression of the phospho-Kin1 mutants was dependent on HDAC6 activity. Expression of Kin1^T/A(8+30)^:GFP resulted in decreased levels of cellular ac-tub, suggesting higher HDAC6 activity in those cells. To test this, tubacin was added to Kin1^T/A(8+30)^:GFP-expressing cells (Figure 6B). Cellular ac-tub levels were restored to levels seen in Kin1:GFP-expressing cells, indicating that Plk1 phosphorylation of Kin1 regulates its ability to inhibit HDAC6 and in turn affect cellular ac-tub levels.

Finally, to address the specificity of the Plk1-dependent phosphorylation of Kin1 in the regulation of ac-tub levels, we determined whether phosphorylation of the Kin1 N-terminus in general affected cellular ac-tub levels. ELM software (http://elm.eu.org) was used to identify potential phosphorylation sites at the N-terminal regulatory region of Kin1 (Supplementary Figure S2C). These sites were mutated to phospho-mimicking mutations, exogenously expressed in cells, and assayed for ac-tub levels (Supplementary Figure S3C). Expression of the phospho-mimicking mutants (other than Kin1^T/E(8+30)^:GFP) showed no changes in cellular ac-tub levels compared with expression of Kin1:GFP.

## Discussion

We had previously shown that depletion of Kin1 results in abnormal mitotic spindles in the form of toppled and monopolar spindles, although the mechanism(s) by which Kin1 regulated spindle formation were not fully understood (Patel et al., 2013). Here we have used live cell imaging to show that in Kin1 depleted cells undergoing mitosis, there was not only a defect in bipolar spindle formation and an increased number of toppled spindles, but also an increased incidence of lagging chromatids. These phenotypes are highly sensitive to MT stability (Palmer et al., 1992) and strongly suggest that the reduced MT stability that we also observed in Kin1 depleted cells contributes to the mitotic phenotypes associated with Kin1 depletion. A well-documented readout of MT stability is its acetylation status, with ac-MTs representing longer-lived MTs (Piperno et al., 1987). In line with Kin1 depleted cells having reduced MT stability, they also have reduced ac-tub levels. HDAC6 is the major cellular tubulin deacetylase (Matsuyama et al., 2002), and inhibition of HDAC6 activity was able to rescue not only ac-tub levels upon Kin1 depletion but also the mitotic spindle defects. Importantly, HDAC6 inhibition also prevented the loss of MT stability seen in the Kin1 depleted cells, indicating that the reduction in ac-tub levels seen in Kin1 depleted cells was not just a marker of reduced MT stability but that the ability of Kin1 to regulate HDAC6 contributes to MT stability. Kin1 may also regulate MT stability indirectly whereby the ability of Kin1 to inhibit HDAC6 may allow regions of MTs to become ‘ac-tub-rich’ and form a platform upon which other proteins that can directly influence MT stability bind and stabilize MTs. These longer-lived MTs would over time attain the acetylation modification. There is some evidence for this, as we observe that Kin1 binds to ac-tub (Supplementary Figure S3A and D) and that the Kin1/HDAC6/Plk1 complex is present on MTs and also sensitive to disruption of the MT cytoskeleton (Figure 5E). This suggests that the MTs (and possibly ac-tub) act as a platform for protein complex formation and signaling.

Although tubulin is a major substrate of HDAC6, it is a multi-domain protein that also catalyzes the deacetylation of a number of other acetylated proteins, acting as an adaptor protein (Valenzuela-Fernandez et al., 2008), and has also recently been shown to have ubiquitination activity (Zhang et al., 2014). Thus, chemical inhibition of HDAC6 by tubacin or its depletion using siRNA as used in this study is likely to impact on multiple pathways and cellular processes. We cannot rule out the possibility that the effects of Kin1 on mitotic spindles are not a direct consequence of tubulin deacetylation by HDAC6 but may result from other HDAC6-dependent mechanisms. However, our data showing Kin1 and HDAC6 co-localization with ac-tub suggest a more direct link between the HDAC6-dependent deacetylation of tubulin and the mitotic defects in Kin1 depleted cells.

Here we show that Kin1 regulates mitosis through its ability to modulate MT stability in an HDAC6-dependent manner and also that Kin1 and HDAC6 exist in a complex at centrosomes and along MTs. Previously, we had shown that Plk1 phosphorylation of Kin1 is required for the ability of Kin1 to regulate mitotic spindle formation and that Kin1 and Plk1 associated and co-localized at centrosomes (Patel et al., 2013). Here we show that Kin1, HDAC6, and Plk1 are all present in a complex together with tubulin and that Plk1 phosphorylation of Kin1 modulates the ability of Kin1 to regulate HDAC6 and cellular ac-tub levels. Our previous studies showed that prolonged (6 h) Plk1 inhibition by BI2536 impairs co-localization of Kin1 and Plk1 at centrosomes (Patel et al., 2013). Under those conditions, it was not possible to determine whether Kin1 and Plk1 association was linked to Plk1 activity or centrosome function, as prolonged Plk1 inhibition disrupts the centrosome cycle. Here short-term inhibition of Plk1 (2 h) using a low concentration (10 nM) of BI2536 does not result in centrosome disruption but impairs the association of both HDAC6 and Plk1 with Kin1. This suggests that the lower ac-tub levels observed upon Kin1 depletion are specific to the Kin1/Plk1/HDAC6 axis but not the centrosome cycle. The reduced association between Kin1 and Plk1 or HDAC6 upon MT disruption also suggests that MTs may act as a platform for the association between Kin1, Plk1, and HDAC6. Thus in cells, both Plk1 activity and the MT cytoskeleton are required for the normal association between Kin1 and HDAC6, Plk1 and MTs.

Much of our focus has been on establishing the role of Kin1, Plk1, and HDAC6 on MT stability during mitosis; however, some of the assays we have used to establish this have been carried out using interphase cells. Plk1 is classically thought to be a mitotic kinase, and thus at first glance, this is somewhat contradictory. Previously, we had shown that endogenous Kin1 interaction and phosphorylation by Plk1 occurs throughout the cell cycle but peaks at G2/M (Patel et al., 2013). Furthermore, Plk1 has been shown to phosphorylate another MT-regulating protein, CLIP^170^, throughout the cell cycle but, as with Plk1 phosphorylation of Kin1, being maximal during mitosis (Li et al., 2010). These data suggest the regulation of Kin1 (and possibly also CLIP^170^) by Plk1 phosphorylation likely plays a role throughout the cell cycle but is most critical during mitosis. There is also now a substantial and accumulating body of evidence that Plk1 plays multiple roles outside of mitosis (Liu et al., 2010).

This novel signaling axis linking Plk1, Kin1, HDAC6 with MT stability has potentially significant consequences in our understanding of KS. KS patients and mice of KS mouse models all present complex pathologies, including skin atrophy (Siegel et al., 2003; Ussar et al., 2008; Rognoni et al., 2014). Here, we show that short-term deletion of Kin1 in the epidermis is associated with reduced levels of ac-tub as well as an increased incidence of toppled (asymmetrical) spindles. Furthermore, this is also associated with reduced proliferation. As reduced ac-tub levels are associated with reduced MT stability and our *in vitro* studies have shown that loss of Kin1 leads to reduced MT stability and ac-tub levels, these data infer that *in vivo* Kin1 also contributes to MT stability during mitosis. Taken together, our data offer a possible mechanism of how Kin1 loss leads to skin atrophy through reduced proliferation due to increased toppled (asymmetrical) cell division and an eventual reduction in the basal proliferative cell population. In our experiments, Kin1 was deleted in the skin for only 10 days and no skin atrophy was observed, which may indicate that Kin1 deletion is required for a longer time for the atrophy to become evident. This is supported by reports in another mouse model in which long-term loss of Kin1 in the skin led to skin atrophy (Rognoni et al., 2014).

In summary, we have identified a novel signaling axis that regulates mitosis involving an adhesion protein (Kin1), a classical cell-cycle regulator (Plk1), and a MT-modifying protein (HDAC6). Their role specifically during mitosis is to regulate MT acetylation and stability at centrosomes and along MTs (Figure 6C). Kin1 binds to HDAC6 directly and can enhance or impair HDAC6 activity depending on its Plk1-dependent phosphorylation status. Thus, using a combination of *in vivo* and *in vitro* approaches, these data provide valuable insight not only into a previously unknown pathway that regulates MT stability and mitosis, but also into how certain aspects of KS, i.e. skin atrophy and reduced cell proliferation, may arise.

## Materials and methods

### Cell culture, transfection, and assay conditions

MDA-MB-231 (American Type Culture Collection) cells were cultured using DMEM (Gibco) supplemented with 10% fetal calf serum (Gibco) and glutamine under standard conditions of 37°C and 5% CO_2_. For short interfering RNA (siRNA) treatments, MDA-MB-231 cells were transfected using 60 nM of siRNA and the recommended Lipofectamine 2000 (Invitrogen) amounts and/or plasmid DNA (0.5–2 μg). All plasmids used were based on pEGFP-N1 backbone (Clontech Laboratories Inc.). All siRNA oligos were purchased from Dharmacon RNAi Technologies. Two siRNA oligos for Kin1 depletion, Kin1 siRNA UTR1 (A004511-14-0005, UCCUUAUCUACAGUUGAUU) and Kin1 siRNA UTR2 (A004511-15-0005, CUAUUAUUUUAGAACCUAG), and non-targeting siRNA (D-001910-0105) were used. For Kin1 siRNA treatment, a pool of UTR1 and UTR2 was used at the final concentration of 60 nM throughout. A pool of siRNA oligos (L-003499-00-0005) was used for HDAC6 knockdown.

The double thymidine block protocol was used to arrest cells at G1/S. Cells were treated with two successive incubations of thymidine (for 17 h and 14 h) 8 h apart. To enrich for mitotic cells, G1/S-arrested cells were released for 6 h. For MT regrowth assays, G1/S-arrested cells were treated for 1 h with nocodazole and then released for 45 min before sample preparation. For tubacin washout assays, cells were treated with tubacin for 16–20 h and then washed and incubated in medium with glutamine and 10% serum for the times indicated. For nocodazole-induced disruption of MTs, G1/S-arrested cells on coverslips were released in the presence of 500 nM nocodazole for 30 min and fixed as described below. For cold-induced disruption of MTs, G1/S-arrested cells were released for 30 min and exposed to 4°C for 20 min and fixed as described below. Tubacin was administered for 16–20 h prior to the endpoint of the experiment. For EG5 inhibitor washout assays, cells were treated with EG5 inhibitor (Tocris, K858) for 2 h before washing three times in medium and incubated in medium with glutamine and 10% serum for the times indicated. Unless stated otherwise, the concentrations of inhibitors used are as follows: BI2536, 10 nM; tubacin, 3 mM; EG5i, 5 μM; nocodazole, 1.6 μM; thymidine, 2 mM.

### Western analysis and immunofluorescence

Cells for western analysis were lysed using NP40 buffer (25 mM Tris, 150 mM NaCl (TBS), 0.5% NP40, pH 7.6) plus protease and phosphatase inhibitors (Roche, 04906845001 and 04693124001, respectively). Proteins were separated according to size using SDS–PAGE, transferred onto nitrocellulose membrane, and blocked for 1 h in TBS + 0.05% Tween-20 (TBST) + 5% BSA. Membranes were incubated for 16 h at 4°C and for 45 min at room temperature for primary and secondary antibodies, respectively. All washes and antibody incubations were carried out in TBST and TBST + 5% BSA, respectively.

For immunofluorescence analysis, cells were grown on glass coverslips, washed once in TBS, and fixed and permeabilized for 10 min at room temperature in Fix Buffer (100 mM PIPES, 3.7% paraformaldehyde, 0.2% Triton X-100, 1 mM MgCl_2_, and 10 mM EGTA, pH 7.4). Coverslips were washed twice in Wash Buffer (TBS plus 0.1% Triton X-100) and incubated with the primary antibody for 16 h at 4°C in Wash Buffer + 2% BSA. Secondary antibody incubations were carried out for 1 h at room temperature in Wash Buffer + 2% BSA. All washes were carried out with Wash Buffer. All coverslips were mounted with Vectashield Mounting Media containing DAPI (Vector Laboratories). Images were captured using an FV-1000 Olympus confocal microscope using a 60× objective.

Antibodies used: anti-Kin1 (Abcam, ab68041, 1:3000 for WB and 1:300 for IF); anti-Plk-1 (Merck Millipore, 05-844, 1:3000 for WB); anti-HDAC6 (CST, 7612); anti-GFP (3999-100); anti-alpha-tubulin (Cell signaling Technology (CST), 2125); anti-acetylated-tubulin (CST, 12152, 1:6000 for WB and 1:600 for IF). Antibodies were used at 1:1000 for western blotting (WB) and 1:200 for immunofluorescence (IF) unless stated otherwise. Actin was visualized with TRITC phalloidin. Dye secondary antibodies for visualizing western blots were used as directed by the manufacturer (Licor). Dye (Alexa Fluor)-conjugated secondary antibodies for visualizing immunofluorescence were used at 1:200.

### GFP pull downs, co-immunoprecipitation, and in vitro binding assays

Sub-confluent cells were washed twice in ice-cold PBS and lysed in NP40 lysis buffer containing protease and phosphatase inhibitors at 4°C. Cell lysate protein (0.25–1 mg) was used for GFP pull downs or immunoprecipitation pull downs. Briefly, the lysate was incubated with agarose beads pre-conjugated with antibodies or ‘GFP Trap’ agarose beads (to pull down GFP) (ChromoTek, gta-20) at room temperature for 30–45 min and washed three times in lysis buffer, once in PBS, and finally again in lysis buffer.

Recombinant HDAC6 (100 ng) and Kin1 fused to GST (GST:Kin1) or GST alone were incubated together in PBS + 0.1% Triton-X 100 (PBST) for 30 min at room temperature. Samples were washed three times in PBST, once in PBS, and then twice in PBST. All samples were boiled for 5 min with sample loading buffer and run out on 4%–15% gradient gels.

### Live cell imaging

MDA-MB-231 cells stably expressing His2B:DsRed were seeded in chambered glass coverslips (Cover glass Lab-tek Chambers, FISHER) and transfected with siRNA oligos 48 h prior to imaging. They were imaged in CO_2_-independent L15 medium (Invitrogen) at 37°C. Chromosome movements were captured every 10 min for 8 h using a 40× 0.85 NA Plan-Apochromat objective lens (Carl Zeiss UK) on a Zeiss Axio-Observer Z1 inverted microscope equipped with a Lumencor Spectra X LED light source (Lumencor Inc.) and a Photometrics Coolsnap HQ_2_ CCD camera (Photometrics). Exposures of 0.1s were applied and every image was captured on five Z planes, 1 micron apart.

### Animals

Mice were generated in which exons 4 and 5 of *Kindlin1 (Fermt1)* were floxed (Kin1^fl/fl^) (Taconics). Kin1^fl/fl^ mice were interbred with K14CreER^T2^ (K14Cre) mice (Li et al., 2000) to give experimental cohorts of K14Cre and K14Cre Kin1^fl/fl^ mice on a FVB/N background. Genotyping was carried out by Transnetyx. All experiments were carried out in accordance with the UK Animal Scientific Procedures Act (1986). Eight-week-old mice were injected with 0.1 mg 4-hydroxytamoxifen (4OHT) (Sigma) for 5 days. After a further 10 days, animals were sacrificed, and dorsal skin was removed and fixed in 10% neutral buffered formalin. Sections were deparaffinized, rehydrated, and unmasked in 10 mM citric acid (pH 6.0) in a pressure cooker for 10 min. They were then blocked with peroxidase (15 min) and protein blocking buffers (2 h) at room temperature (DakoCytomation). Sections were incubated overnight at 4°C with primary antibodies diluted in Antibody Diluent, washed 5 times in 0.1% Tween-20/TBS (TBST), and incubated with Alexa Fluor-488-conjugated secondary antibody (Invitrogen) for 1 h at room temperature. Sections were rinsed 5 times in TBS and mounted with Vectashield Mounting Media containing DAPI (Vector Laboratories). The following antibody dilutions were used: anti-alpha-tubulin, 1:300 (CST, 2125) and anti-acetylated-tubulin, 1:800 (CST, 12152)

## Supplementary material

Supplementary material is available at *Journal of Molecular Cell Biology* online.

## Funding

This work was supported by funding from ERC (294440), CRUK (C157/A15703), and DEBRA (Brunton 1).

**Conflict of interest:** none declared.

## Supplementary Material

Supplementary Data
