# Supplementary material for: Kindlin1 regulates microtubule function to ensure normal mitosis
Source: J Mol Cell Biol. 2016 Aug 19;8(4):338–48. doi: 10.1093/jmcb/mjw009 (PMC4991666; doi:10.1093/jmcb/mjw009)
Supplement: Supplementary Data [file supp_8_4_338__index.html]

Kindlin1 regulates microtubule function to ensure normal mitosis — Kindlin1 regulates microtubule function to ensure normal mitosis — Supplementary Data 

# Kindlin1 regulates microtubule function to ensure normal mitosis

## Supplementary Data

Supplementary Data

- Supplementary Data - Pdf file
- Supplementary Video 1 - avi file
- Supplementary Video 2 - avi file
- Supplementary Video 3 - avi file
