# Supplementary material for: Kindlin1 regulates microtubule function to ensure normal mitosis
Source: J Mol Cell Biol. 2016 Aug 19;8(4):338–48. doi: 10.1093/jmcb/mjw009 (PMC4991666; doi:10.1093/jmcb/mjw009)
Supplement: Supplementary Data [file supp_mjw009_mjw009supp.pdf]

Supplementary Figure S1

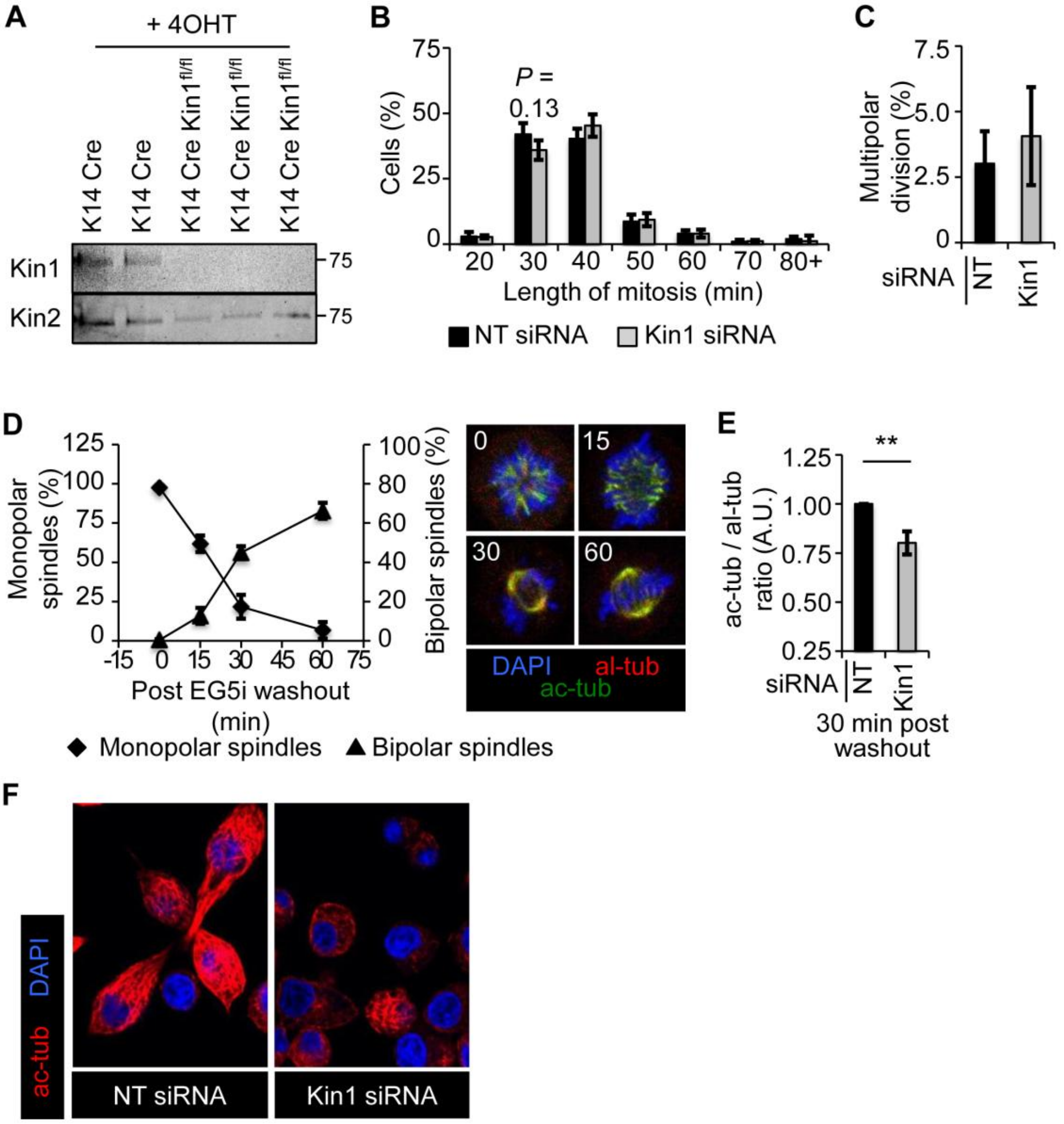

Supplementary Figure S1 (A) Western analysis confirming Kin1 but not Kin2 is deleted in keratinocytes. (B-C) Quantification of live cell imaging of control (black bars) and Kin1 depleted (grey bars) cells undergoing mitosis. (B) Length of mitosis from nuclear envelope breakdown to anaphase onset. (C) The incidence of multipolar spindles at metaphase-anaphase transition (F-G) EG5 inhibitor (EG5i) washout assays (see Materials). (D) Quantification of monopolar and bipolar spindles after release from EG5 inhibition of control cells (left). Typical images of the spindles observed at

the time points indicated after release from EG5 inhibition (right). (E) The ratio in Kin1 depleted and control cells 30 min after EG5i washout. (F) Representative images used in the quantification of ac-tub levels following Kin1 depletion in cells. In all graphs shown, n ≥ 3, error bars are ± s.e.m. and the significance (p values, t-test) shown above (* < 0.05 and ** < 0.01).

Supplementary Figure S2

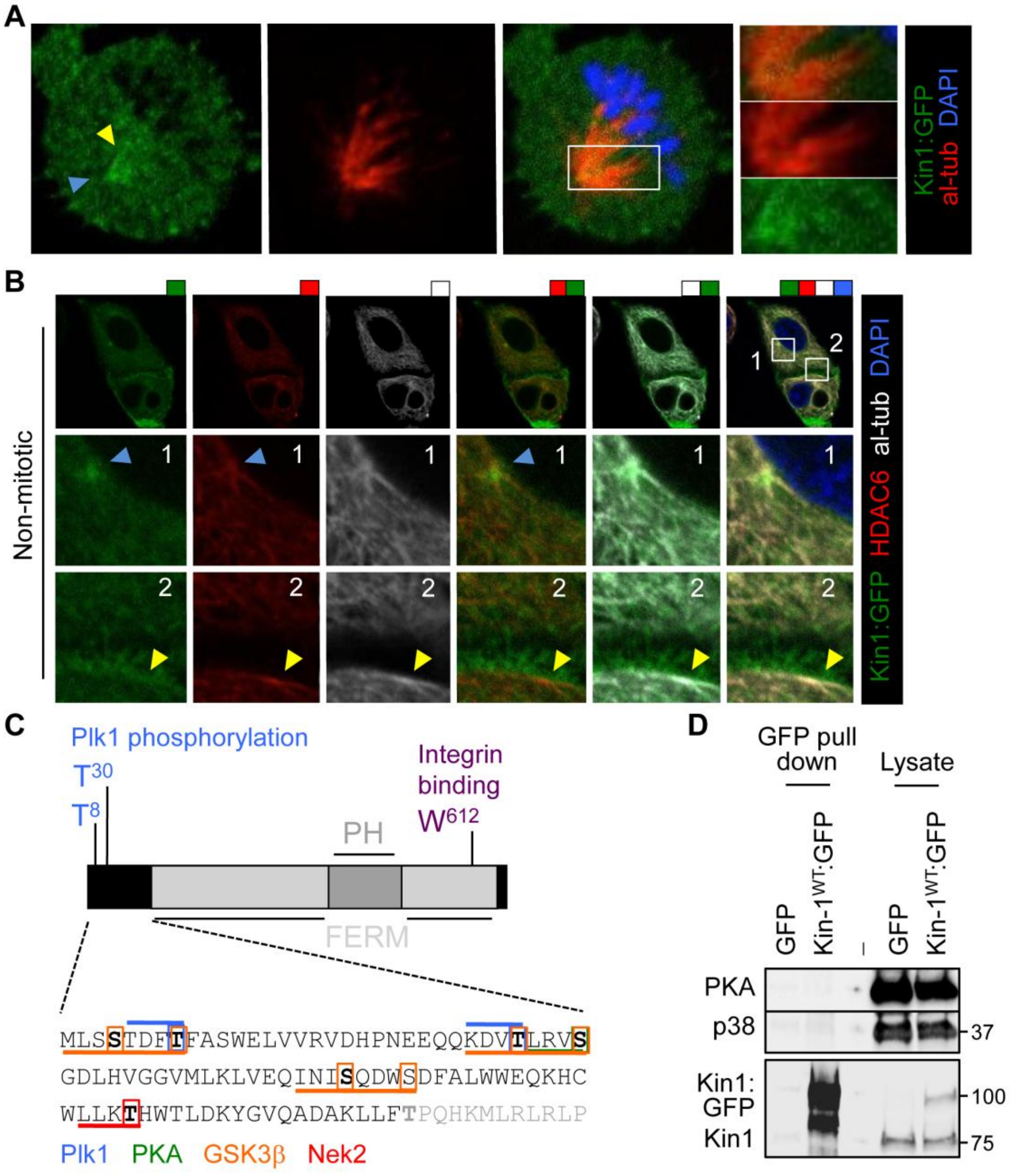

Supplementary Figure S2 Localization of Kin1:GFP in mitotic (A) and non-mitotic (B) cells at centrosomes (blue arrowhead) and along MTs (yellow arrowhead). (B) An example of HDAC6, Kin1:GFP and al-tub co-localization at the centrosome (blue arrowhead) and along MTs (yellow arrowhead) in a non-mitotic cell. (C) Top, a schematic diagram of the domain structure of Kin1 with key residues indicated. Middle, the amino acid sequence of the potential regulatory N-terminus prior to the FERM and PH domains. Known (T8 and T30) and potential phosphorylation sites (S4, S34, S53 and S73) that may undergo phosphorylation are indicated (boxed), the consensus site (underlined) and the potential kinases that may be responsible (below) are shown. (D) GFP pull down from protein lysate of cells expressing either Kin1:GFP or GFP.

Supplementary Figure S3

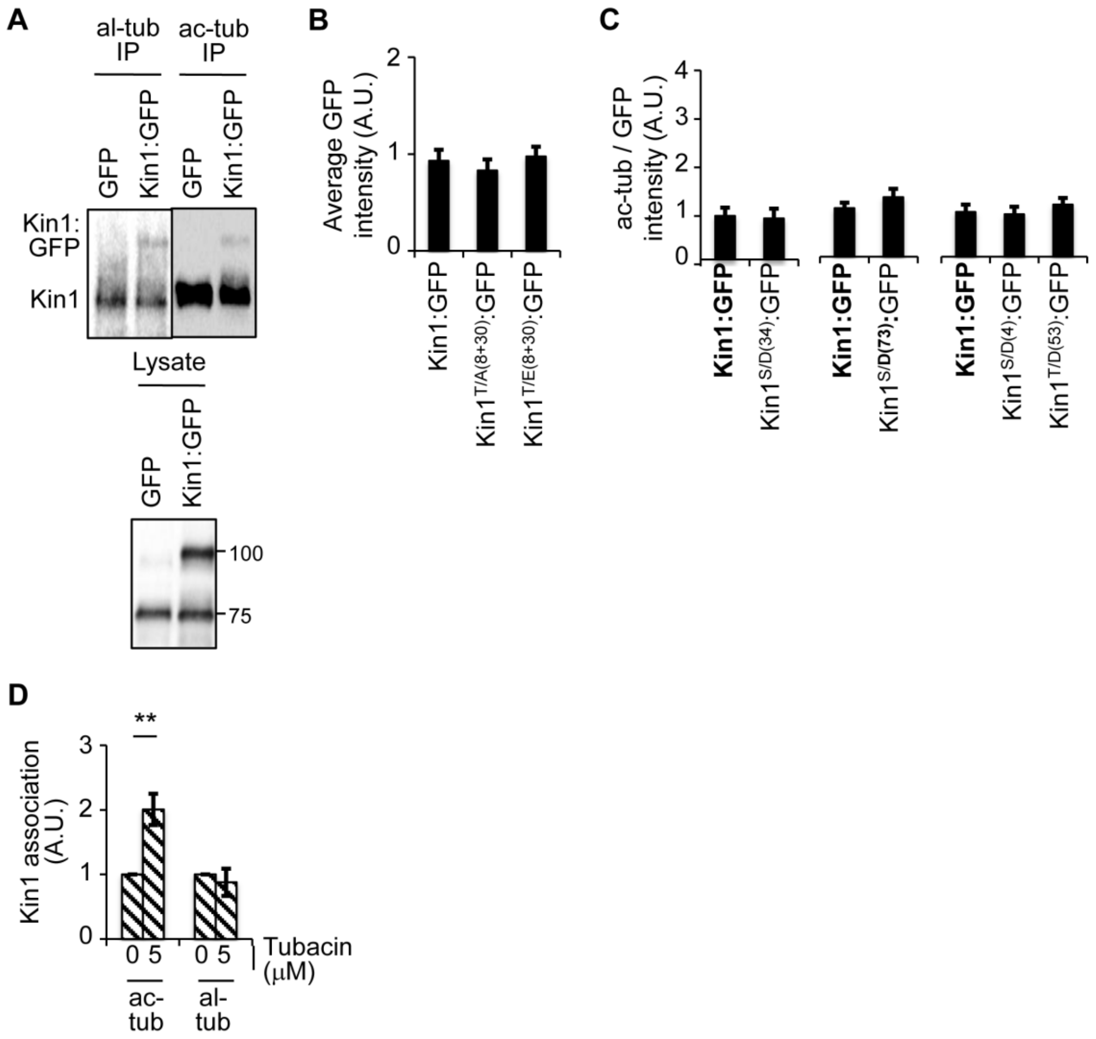

Supplementary Figure S3 (A) Co-IPs using al-tub and ac-tub specific antibodies. (B) The average fluorescence intensity of cells expressing Kin1:GFP, Kin1$^{T/A(8+30)}$:GFP and Kin1 $^{T/E(8+30)}$:GFP. (C) Ac-tub levels (MT-regrowth assays) are unchanged upon expression of phospho-mimicking mutants of the potential phosphorylation sites highlighted in Supplemental Figure 2C. (B and C) > 300 cells were measured. (D) Quantification of western analysis of Kin1:GFP association with ac-tub and al-tub ± tubacin. In all graphs shown, $n \geq 3$, error bars are ± s.e.m. and the significance (p values, t-test) shown above (* < 0.05 and ** < 0.01).

Supplementary Movie S1

Example of an NT siRNA treated (control) cell stably expressing His2B:DsRed undergoing mitosis.

Supplementary Movie S2

Example of a Kin1 siRNA treated (Kin1 depleted) cell stably expressing His2B:DsRed undergoing toppled mitosis.

Supplementary Movie S3

Example of a Kin1 siRNA treated cell (Kin1 depleted) stably expressing His2B:DsRed undergoing mitosis where lagging chromatids were present.
